# The Menstrual Endometrium: From Physiology to Future Treatments

**DOI:** 10.3389/frph.2021.794352

**Published:** 2022-01-31

**Authors:** Marianne Watters, Rocío Martínez-Aguilar, Jacqueline A. Maybin

**Affiliations:** ^1^Simpson Centre for Reproductive Health, Royal Infirmary of Edinburgh, NHS Lothian, Edinburgh, United Kingdom; ^2^MRC Centre for Reproductive Health, University of Edinburgh, Edinburgh, United Kingdom

**Keywords:** menstruation, endometrial, adenomyosis, abnormal uterine bleeding, inflammation, coagulation, vascular, hypoxia

## Abstract

Abnormal uterine bleeding (AUB) is experienced by up to a third of women of reproductive age. It can cause anaemia and often results in decreased quality of life. A range of medical and surgical treatments are available but are associated with side effects and variable effectiveness. To improve the lives of those suffering from menstrual disorders, delineation of endometrial physiology is required. This allows an increased understanding of how this physiology may be disturbed, leading to uterine pathologies. In this way, more specific preventative and therapeutic strategies may be developed to personalise management of this common symptom. In this review, the impact of AUB globally is outlined, alongside the urgent clinical need for improved medical treatments. Current knowledge of endometrial physiology at menstruation is discussed, focusing on endocrine regulation of menstruation and local endometrial inflammation, tissue breakdown, hypoxia and endometrial repair. The contribution of the specialised endometrial vasculature and coagulation system during menstruation is highlighted. What is known regarding aberrations in endometrial physiology that result in AUB is discussed, with a focus on endometrial disorders (AUB-E) and adenomyosis (AUB-A). Gaps in existing knowledge and areas for future research are signposted throughout, with a focus on potential translational benefits for those experiencing abnormal uterine bleeding. Personalisation of treatment strategies for menstrual disorders is then examined, considering genetic, environmental and demographic characteristics of individuals to optimise their clinical management. Finally, an ideal model of future management of AUB is proposed. This would involve targeted diagnosis of specific endometrial aberrations in individuals, in the context of holistic medicine and with due consideration of personal circumstances and preferences.

## Introduction

The symptom of abnormal uterine bleeding (AUB) is defined by the International Federation of Gynecology and Obstetrics (FIGO) as bleeding from the uterine corpus that is outside the normal parameters defined by AUB System 1 (Table 1) for duration, volume, frequency and/or regularity (1, 2). It encompasses heavy menstrual bleeding (HMB) and intermenstrual bleeding. AUB becomes chronic when symptoms are present for the majority of the preceding 6 months.

**Table 1:** International Federation of Gynaecology and Obstetrics (FIGO) Abnormal Uterine Bleeding System 1 definitions for normal menstrual bleeding [(2). doi: 10.1002/ijgo.12666].

**Parameter**	**Normal**
Frequency	24–38 days
Duration	Up to 8 days
Regularity	Regular variation (shortest to longest ≤ 9 days)
Flow volume	Normal
Intermenstrual bleeding (bleeding between the cyclically regular onset of menses)	None
Unscheduled bleeding on progestins ± oestrogen	Not applicable if not on hormonal medication or none

AUB is a symptom and not a diagnosis. The FIGO AUB System 2 PALM-COEIN provides a system for diagnosing the underlying pathology resulting in the symptom of AUB (1, 2). PALM encompasses structural disorders such as polyps (AUB-P), adenomyosis (AUB-A), leiomyoma (AUB-L) and malignancies (AUB-M). COEIN refers to non-structural causes including coagulopathies (AUB-C), ovulatory dysfunction (AUB-O), endometrial disorders (AUB-E), iatrogenic causes (AUB-I) and not otherwise classified (AUB-N).

Approximately one third of women of reproductive age experience AUB at some point in their reproductive lives (3). This translates to approximately 600 million women worldwide with debilitating symptoms that negatively impact their quality of life (4). In the UK, over 800,000 women seek medical help for AUB annually and it is the fourth most common referral to UK gynaecological services (5). The impact amongst those who do not present for medical review is unquantifiable. It is thought that many women endure years of AUB that may result in anaemia, affect mental health and result in financial hardship (4).

There are a range of current medical treatments for AUB, but these are often limited by lack of effectiveness and intolerable side effects. Hormonal medications are the mainstay of medical management for AUB. These preparations act to override physiological ovarian hormone production and do not specifically target the underlying cause of AUB in many cases. Hormonal medications have a range of contraindications and side effects and are particularly unsuitable for women who wish to conceive. Most importantly, women with AUB often find these treatments ineffective meaning that up to 60% of these women resort to fertility-ending surgical procedures with associated surgical risk (5). To improve medical strategies for AUB it is important to understand endometrial physiology and accurately diagnose the aberrations that result in AUB.

This review examines our current knowledge of menstrual physiology and the key processes involved. Aberrations which lead to AUB will be discussed, with a focus on AUB-E as an example of a non-structural cause and AUB-A as a structural diagnosis. Reference is also made to AUB-O, as most of the current treatments for AUB act at the ovarian level. However, detailed review of the processes and subsequent management of AUB –P/L/M/C/O/I/N is not included and we signpost readers to existing comprehensive reviews covering AUB-L (6), AUB -M (7) and AUB-C (8). Current management of AUB-A and AUB-E and its limitations will be reviewed, followed by discussion of recent advances and potential new therapeutic targets. Finally, a model of future management of AUB will be proposed with the aim of providing more effective, personalised treatments for those who are suffering with this debilitating symptom.

## Physiology of Menstruation

The endometrium forms the inner lining of the uterus and requires an ability to change across the menstrual cycle to regenerate, decidualise, shed and to support implantation and pregnancy when necessary. In the absence of implantation, shedding of the luminal two-thirds of the endometrium occurs in a process known as menstruation. This occurs under the strict control of endocrine, immune, vascular and coagulation systems.

### Physiology: Endocrine Regulation

The endometrium is a complex and dynamic multicellular tissue that responds to the ovarian hormones. Oestradiol is most abundant in the first half of the menstrual cycle, coincident with high rates of endometrial cell proliferation (9). Following ovulation, the endometrial secretory phase commences, where high levels of progesterone produced by the *corpus luteum* lead to altered endometrial morphology to prepare for implantation. If implantation does not occur, the *corpus luteum* regresses. Subsequent withdrawal of progesterone and oestradiol triggers a series of molecular and cellular events that resemble a classical inflammatory episode (pain, heat, redness and swelling) (10) and culminates with menstruation.

### Physiology: Endometrial Breakdown and Inflammation

The progesterone withdrawal that occurs during the late secretory phase releases the transcription factor nuclear factor kappa B (NFκB) from its association with inhibitory proteins, such as IκB (11, 12). Once free, NFκB translocates to the nucleus, where it enhances the expression of inflammatory mediators (13, 14) including cytokines [tumour necrosis factor (TNF), interleukin-6 (IL6)] and chemokines [C-C motif chemokine ligand 2 (CCL2), interleukin-8 (CXCL8)] (13, 14). These mediators promote specific leukocyte trafficking and recruitment of myeloid cells (15). Activation of the NFκB pathway has been immunohistochemically detected in the endothelial, glandular and stromal compartments of the secretory endometrium (11). However, a thorough delineation of the particular cells types responsible for the initiation of the inflammatory cascade at menstruation is still lacking.

More recently, the effects of the inflammasome on the release of inflammatory cytokines during menses has been described, *in vitro* and *ex vivo* (16). The inflammasome is a multiprotein assembly that is classically associated with inflammatory signalling amplification (17). Both the NFκB and inflammasome systems may act simultaneously at menses to recruit immune cells to the endometrium. During menses, the most prevalent myeloid cells present in endometrial tissue are neutrophils and macrophages (18). Both myeloid cells activate and release matrix metalloproteinases (MMPs) in the endometrial milieu. MMPs are widely accepted as being responsible for the shedding of the upper layers of the endometrium during menses (18), although the contribution of reactive oxygen species has also been suggested (19).

Once endometrial shedding has been accomplished, the inflammatory events which led to tissue destruction must be controlled in order to allow repair processes to begin. Several anti-inflammatory mediators emerge as candidates responsible for limiting the inflammatory response, including the glucocorticoid cortisol and lipid mediators.

Exposure of epithelial ovarian cells to the pro-inflammatory cytokine interleukin-1α (IL1A) has been found to increase hydroxysteroid 11-β dehydrogenase 1 mRNA (*HSD11B1*) (20). *HSD11B1* catalyses the final step of cortisol synthesis, regulating the availability of this anti-inflammatory steroid. Similar regulation may be present in the menstrual endometrium, where the pro-inflammatory environment may activate anti-inflammatory pathways to resolve inflammation. Indeed, *HSD11B1* mRNA was found to be increased in the endometrium at the time of menses (21), consistent with cortisol having a role in menstrual inflammatory resolution. A local increase in endometrial cortisol levels at menstruation may also result in a pro-repair environment. *In vitro* treatment of human endometrial stromal cells with cortisol was found to increase active transforming growth factor β (TGFB) in cell culture supernatants (22), an ambivalent soluble mediator with context-dependent pro-inflammatory or restorative properties (23). Cortisol has also been shown to affect the macrophage secretome, with supernatant from cortisol-treated peripheral blood monocyte-derived macrophages resulting in altered endometrial endothelial cell expression of angiogenic genes C-X-C motif chemokine ligand 2 (*CXCL2*), *CXCL8*, connective tissue growth factor (*CCN2*), and vascular endothelial growth factor C (*VEGFC)* with a putative role in vascular repair (24). Cortisol has also been involved in the regulation of the platelet factor 4 (CXCL4/PF4) released by endometrial cells *in vitro*. This factor may be involved in endometrial repair by promoting the recruitment of reparative macrophages (25). Therefore, cortisol may limit the inflammatory response and create a pro-repair endometrial environment.

The presence of lipid mediators has also been associated with the resolution of inflammation. Specifically, lipoxins are lipid mediators with anti-inflammatory and pro-resolution properties that are present systemically (26). During menses, the lipoxin A4 receptor was increased in the endometrium at the mRNA level (27). Furthermore, *in vitro* studies showed that addition of lipoxin A4 to endometrial explants primed with an inflammatory stimulus mitigates the subsequent pro-inflammatory response (27). Hence, lipoxin A4 and other lipid mediators may play a role in limiting the inflammatory response within the menstrual endometrium and merit further study.

### Physiology: Limiting Blood Loss

In addition to the resolution of menstrual inflammation, further mechanisms exist to limit menstrual blood loss. NFκB activation promotes the expression of prostaglandins and enzymes involved in their synthesis, such as cyclooxygenase-2 (COX2/PTGS2). While COX2 plays a role in endometrial breakdown (28, 29), prostaglandin F2α (PGF2α) (30), along with other vasoconstrictors like endothelin-1 (EDN1) (31), may curtail menstrual blood loss by constricting endometrial arterioles. Haemostatic mechanisms are also required to limit menstrual blood loss (8). During primary haemostasis, platelets adhere to the injured vascular endothelium and interact with the surrounding matrix, creating a platelet plug. The resulting platelet aggregation triggers activation of the coagulation system which, through complex interactions, converts soluble fibrinogen into an insoluble fibrin clot (32). In the endometrium, pre-clinical studies predict that platelet aggregation events are less crucial than vasoconstriction and fibrin clot formation (33). However, both the dysregulation of platelet aggregation and/or fibrin clot formation may have a negative impact upon menstrual blood loss (8).

### Physiology: Endometrial Repair and Regeneration

After endometrial shedding, the denuded surface needs to be restored to minimise blood loss and recover its functionality for the next cycle. Endometrial hypoxia has been proposed as an important regulator of endometrial repair. Intensive vasoconstriction of the spiral arterioles during menstruation was directly observed in endometrial explants transplanted into the anterior chamber of the eye of rhesus monkeys by Markee in 1940 (34). More recently, markers of endometrial hypoxia have been detected in both in pre-clinical models (35–37) and the human endometrium (38, 39) during menses.

Although hypoxia does not appear to be required for endometrial breakdown (40), it may be important for triggering menstrual endometrial repair (37). Hypoxia inducible factor (HIF), is composed of an oxygen regulated alpha subunit (HIF1A) and a constitutively expressed beta subunit to form a transcription factor responsible for the cellular adaptative response to hypoxia (41). It is proposed that HIF1A is required for normal endometrial repair during menstruation, due to its exclusive presence in the perimenstrual phase, alongside evidence of delayed endometrial repair during menstruation with genetic or pharmacological reduction of HIF1A in mouse studies (37). HIF1A enhances the endometrial transcription of several genes involved in endometrial repair and blood vessel formation such as adrenomedullin, *CCN2, CXCL8* and *VEGF* (42–44). Interestingly, some of these mediators can also be synergistically upregulated via prostaglandin action (44), which may represent dual regulation to ensure timely repair and cessation of menstrual bleeding.

## Pathology of Menstruation

As described above, menstruation relies on meticulously coordinated endocrine, immune, vascular and haemostatic responses to limit blood loss and ensure optimal repair. Thus, repression or overactivation of the biochemical pathways involved in this process may result in pathological manifestations. The role of (i) endocrine regulation, (ii) tissue breakdown and inflammation, (iii) vascular function and coagulation and (iv) endometrial repair in AUB is discussed below, with a focus on AUB-E and AUB-A.

AUB-E is a non-structural cause of AUB and is diagnosed when other causes of AUB have been excluded clinically. AUB-E represents an under-researched area where precise mechanisms resulting in this particular subtype of AUB remain undefined. AUB-A is an example of a structural cause of AUB. Adenomyosis develops a result of endometrium or endometrial-like tissue being present within the myometrial layer of the uterus. Adenomyosis may be asymptomatic or may cause symptoms such as dysmenorrhoea, subfertility or AUB (AUB-A) (45). Traditionally, adenomyosis has been diagnosed retrospectively following hysterectomy. There now is evidence to support ultrasound diagnosis of adenomyosis (46) but there remains a pressing clinical need to improve our understanding of the mechanisms causing AUB-A to improve diagnosis and management.

### Pathology: Endocrine Regulation

Aberrant endocrine regulation of the endometrium is not known to be present in AUB-E or AUB-A but does occur due to ovulatory dysfunction (AUB-O). Menstrual disturbance in AUB-O occurs due to persistence of oestradiol signalling and lack of *corpus luteum* formation (47). The resulting lack of progesterone and subsequent progesterone withdrawal may result in heavy, infrequent and/or irregular menstrual bleeding. AUB-O occurs frequently at menarche and during peri-menopause or in those with polycystic ovary syndrome. Many current treatments for AUB act to override physiological ovarian hormone production and are often helpful in AUB-O. However, as endocrine dysregulation is rarely the primary cause of AUB-E and AUB-A, treatment failures occur and are discussed in further detail below.

### Pathology: Endometrial Breakdown and Inflammation

Patients with AUB-E have been shown to have higher levels of TNF protein in their menstrual effluent when compared to those with normal menstrual blood loss (NMB) (48). TNF is a downstream inflammatory target in NFκB signalling, which can also act as an NFκB inducer (49). COX is another NFκB downstream inflammatory effector that is dysregulated in AUB. This biosynthetic enzyme possesses two isoforms (COX1/PTGS1 and COX2/PTGS2) and the mRNA of both were found to be increased in endometrium from those with AUB-E (50). This supports the hypothesis that excessive endometrial inflammation may be one mechanism causing AUB-E.

In the early stages of endometrial breakdown, local inflammation is tightly controlled. It may be hypothesised that those with AUB-E have disproportionate endometrial recruitment of neutrophils and macrophages that generate an inflammatory positive loop, where more mediators are released and further myeloid cells are recruited. In turn, these cells may over activate the secretion and release of MMPs leading to excessive or prolonged endometrial breakdown. Mechanistic preclinical studies focusing on the impact that upregulation of TNF, COX and other NFκB-induced inflammatory mediators have on endometrial leukocyte recruitment and MMPs activation are needed to confirm or refute this hypothesis.

An altered inflammatory response may also contribute to the symptom of AUB-A. In the presence of adenomyosis, NFκB binding activity is constitutively overactivated both in the eutopic endometrium (51) and adenomyotic lesions (51, 52). The increase in inflammatory cytokines released by leukocytes isolated from both the eutopic endometrium and adenomyotic lesions of women with adenomyosis compared to healthy controls (53) also suggests inflammatory dysregulation. Moreover, NFκB activity has been positively associated with the symptom of AUB-A (54). Interestingly, in those with AUB-A, *COX2/PTGS2* mRNA levels both in the adenomyotic lesions and the eutopic endometrium are increased compared to adenomyotic patients with NMB. This *COX2/PTGS2* increase correlated with higher expression of the pro-inflammatory mediators *IL6* and *CXCL8* (55), consistent with a pro-inflammatory endometrial environment increasing menstrual blood loss in those with adenomyosis.

As previously discussed, endometrial cortisol may play a role the resolution of the menstrual inflammatory response. Patients with AUB-E have been shown to have an increased endometrial expression of the cortisol-inactivating enzyme hydroxysteroid 11-β dehydrogenase 2 (*HSD11B2*) (56) as well as a decrease in the downstream cortisol target CXCL4/PF4 during menses (25). Hence, cortisol deficiency may play a key role in AUB-E. At present, there are no studies exploring a potential correlation between cortisol levels and AUB-A.

### Pathology: Limiting Blood Loss

As described above, arteriole vasoconstriction is a key process to limit menstrual blood loss. Defective vasoconstriction may have its origin in alterations in vasoconstrictive mediators. In AUB-E, a decrease in the vasoconstrictor EDN1 at the protein level has been described, as well as an increase in the enzyme responsible for its inactivation, neutral endopeptidase (57). There is also evidence of higher endometrial levels of the vasodilating prostaglandin PGE_2_ in those with HMB compared to NMB (50). These effects in conjunction with decreased mRNA levels of the PGF2α receptor (*PTGFR*) (58), cause a reduction in the ratio PGF2α/PGE_2_ which may result in a defective ability to limit menstrual blood loss (50, 58).

In patients with AUB-A, there are no reports of endometrial differences in EDN1 or prostaglandins. Studies are required to examine the eutopic endometrium in those with AUB-A and control groups of those with adenomyosis who do not experience AUB and those free from disease and symptoms. However, within adenomyotic lesions, the vasoconstrictor/vasodilator balance appears to be disrupted. Preliminary data from *in vitro* studies (59) show that cells from adenomyotic lesions have higher mRNA concentrations of prostaglandin E synthase 2 (*PTGES2*)- the enzyme responsible of PGE_2_ synthesis- compared to eutopic endometrial cells from those without adenomyosis (59). In a pre-clinical mouse model, prostaglandin D2 genetic deficiency increased endometrial COX2/PTGS2 and PGE_2_ levels and increased adenomyosis lesion development (60). Therefore, an imbalance of vasoconstrictor and vasodilator molecules may be involved in the development of adenomyotic lesions but its impact on the development of AUB remains to be determined.

AUB may additionally or alternatively result from aberrant endometrial haemostatic processes, such as fibrin clot formation/degradation ratios. Patients with AUB-E were found to have increased activity of the tissue plasminogen activator (PLAT), compared to those with NMB (61). This mediator activates plasminogen, which is the enzyme responsible for the degradation of fibrin clots. In contrast, the eutopic endometrium and lesions of those with adenomyosis were found to have higher levels of the plasminogen activator inhibitor 1 (SERPINE1) -a PLAT inhibitor-when compared to healthy controls (62). Whether such aberrations result in less fibrinolysis or represent compensatory mechanisms remains to be determined.

Defects in primary haemostasis might also be involved in AUB-A. Tissue factor (F3), a protein involved in the initiation of the coagulation cascade, was found to be increased in the eutopic endometrium as well as in the lesions of adenomyotic patients when compared to healthy controls (63). Interestingly, F3 endometrial immunohistochemical staining of glandular epithelial cells was significantly higher in women with AUB-A than those adenomyosis patients with normal menses (63). To determine the role of haemostasis dysregulation in AUB-A, combining a pre-clinical mouse model of adenomyosis (64) with simulated menstruation (36, 37) would allow genetic and/or pharmacological alteration of the platelet cascade to examine the impact on menstrual blood loss/endometrial repair.

### Pathology: Endometrial Repair and Regeneration

Defective vasoconstriction may also affect endometrial hypoxia and menstrual endometrial repair. Patients with AUB-E have been shown to have decreased menstrual endometrial HIF1A protein when compared to those with NMB, as well as a reduction in HIF1 downstream targets (37). In a mouse model of simulated menses, *HIF1A* genetic deficiency or HIF1 pharmacological inhibition, delayed endometrial repair (37). However, specific cell types driving these HIF1A mediated effects in the menstrual endometrium remain undefined. Those with AUB-E have also been shown to have menstrual deficiencies in other putative endometrial repair factors, with lower protein levels of TGFB perimenstrually when compared to those with NMB (22).

The decreased bioavailability of HIF1A and TGFB in patients with AUB-E may affect vascular repair and/or angiogenesis after endometrial shedding (65). Defects in spiral arteriole maturation have been described in AUB. Patients with AUB-E display greater focal discontinuities in endometrial blood vessel walls than those experiencing NMB (66). A lower proliferation rate of vascular smooth muscle cells (VSMCs) in spiral arterioles of those with AUB-E has also been demonstrated and these cells appear critical for vessel integrity and blood flow (67). In addition, VSMCs in the spiral arterioles of AUB-E patients exhibit a lower expression of maturation markers [alpha smooth muscle actin (ACTA2), myosin heavy chain (MYH)] (68). Moreover, some components of the endometrial endothelial extracellular matrix [laminin (LAM), osteopontin (SPP1), fibronectin (FN1), collagen IV (COL4)] are dysregulated in AUB-E, which may contribute to reduced endothelial vascular integrity (69).

Patients with adenomyosis have also been shown to have evidence of abnormal endometrial vascularisation. The microvascular density of both the eutopic endometrium and adenomyotic lesions was higher than in the endometrium of healthy controls (70, 71). Furthermore, VEGF protein levels show a similar trend, being higher in eutopic endometrium (71, 72) and lesions of adenomyotic patients (52, 70, 73) when compared to healthy controls. None of these studies quantified menstrual blood loss or considered the presence of the symptom of AUB in their analysis. This is necessary to determine if abnormal endometrial vascularisation is causing the symptom of AUB and/or is involved in the development of adenomyotic lesions.

## Current Treatment

There are a range of medical treatments currently available for the treatment of AUB. This section discusses these options in the context of the menstrual physiological processes on which they exert their actions.

### Current Treatment: Endocrine Manipulation

Most current medical treatments for AUB act to override physiological ovarian hormone production. As discussed, however, there are multiple pathways involved in the pathogenesis of AUB-E and AUB-A and endocrine manipulation fails to directly target these processes. Hormonal medication may be more suitable for those with AUB-O where aberrant hormone secretion and regulation leads to AUB symptoms. Despite this, medical therapy in the form of the combined oral contraceptive pill (COCP), oral progestins and levonorgestrel-releasing intrauterine system (LNG-IUS) are commonly offered to patients who present with AUB as first line treatment (74). The COCP has been shown to be effective in reducing HMB and regulating unscheduled bleeding (75) but it is not suitable for those with a history of migraine with aura (sensory disturbance accompanying migraine symptoms), a personal or strong family history of venous thromboembolism, a body mass index (BMI) > 35, smokers over the age of 35 or those wishing to conceive (76). Side effects such as mood changes, skin changes and fluctuation in weight are reported. Oral progestins are available to a larger group of patients, with fewer contraindications, but again these medications come with similar reported side effects and a negative impact on conception which may render them unsuitable for certain populations.

Interestingly, of the hormonal treatments outlined above, only the LNG-IUS has been shown to improve quality of life (77) and there is evidence that it improves HMB (78). A randomised control trial comparing the LNG-IUS and COCPs for symptomatic treatment of adenomyosis, showed the LNG-IUS to be more effective in reducing pain and bleeding (79). However, some women may find IUS insertion painful and the risks of uterine perforation or infection may be unacceptable. It may also cause unpredictable bleeding patterns and hormonal side effects of acne, breast tenderness and mood changes (80).

Gonadotropin releasing hormone (GnRH)-agonists may also be used for AUB management (81, 82). The sustained activation of GnRH receptors leads to their desensitisation, ultimately inhibiting luteinizing hormone (LH) and follicle-stimulating hormone (FSH) synthesis by the pituitary gland. Abrogation of these hormones suppresses ovulation and consequently oestrogen (oestradiol) production. This ovarian hormonal suppression also usually results in the absence of menstruation. However, one of the main downsides of this treatment is the negative side effects caused by oestrogen deficiency. In addition to hot flushes and/or loss of libido, patients may experience a reduction in bone mineral density, increasing the risk of osteoporosis (83). Therefore, its use is limited in younger women. If used in young women, hormone replacement therapy (HRT) is recommended to reduce menopausal symptoms and risk of loss of bone density.

### Current Treatment: Breakdown and Inflammation

More specific correction of the aberrations present in those experiencing menstrual disorders is currently possible. Non-hormonal medical treatment includes the use of non-steroidal anti-inflammatories (NSAIDs). NSAIDs target the excessive endometrial inflammation observed in AUB-E (48) by inhibiting the COX enzymes that are responsible for the synthesis of prostaglandins (28, 29). The ability of NSAIDs to reduce menstrual blood loss is highlighted in a meta-analysis of 18 randomised controlled trials which demonstrates that NSAIDs are more effective in reducing menstrual blood loss when compared with placebo (84). Mefenamic acid and naproxen have both been shown to result in a reduction in levels of menstrual blood loss, with no significant difference noted between the two (85, 86). NSAIDs use may be limited in patients with a history of gastrointestinal bleeding, inflammatory bowel disease, severe asthma, renal disease, congestive heart failure and cerebrovascular disease. NSAIDs can also affect platelet function and when used in individuals with underlying coagulopathies NSAIDs may be ineffective and may lead to increased bleeding (87).

### Current Treatment: Limiting Blood Loss

As discussed, a key process involved in limiting menstrual blood loss is primary haemostasis and the creation of a platelet plug. This triggers a series of interactions resulting in conversion of soluble fibrinogen to an insoluble fibrin clot. Tranexamic acid (TXA) is a medication which may be used to reduce the breakdown of the fibrin clot. TXA competitively blocks plasminogen binding sites and reduces the production of plasma and the breakdown of fibrin. In a meta-analysis, which predominantly incorporated results from studies in patients with AUB-E, tranexamic acid was demonstrated to be superior to placebo in reducing menstrual blood loss (88). It has fewer contraindications when compared with hormonal therapies but its use is limited in patients with a history of thromboembolic disease (76).

## Emerging Treatments

**Figure 1:**
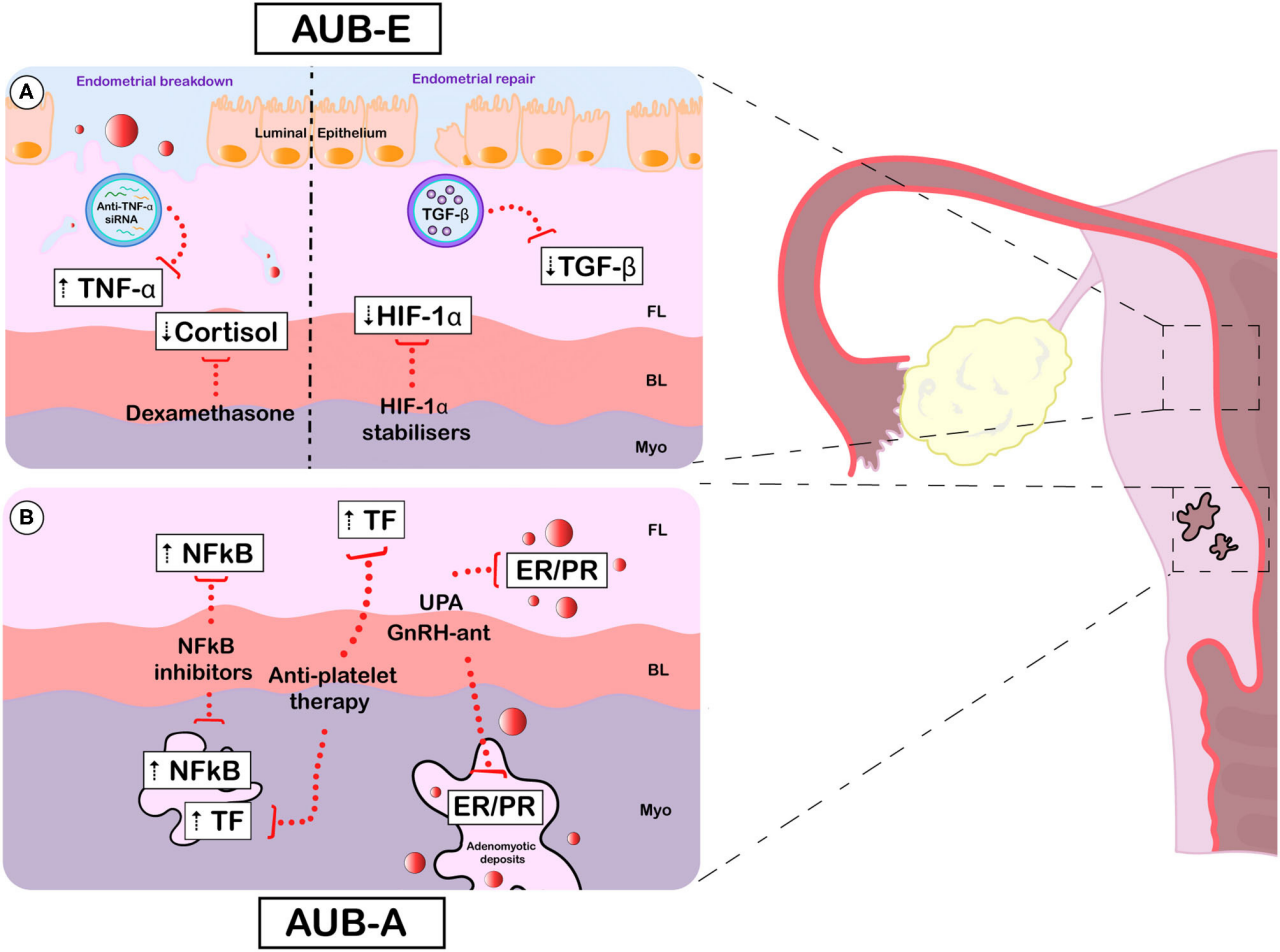
Emerging and potential future treatments for AUB-E and AUB-A. Specific aberrations (white boxes) and emerging therapeutic targets (red dotted lines) for AUB-E **(A)** and AUB-A **(B)**. [**(A)**, left] During active endometrial breakdown, the TNF upregulation observed in AUB-E could be targeted using nanoparticles loaded with small interfering RNA against TNF. Cortisol deficiency at menses may be restored by administration of the glucocorticoid dexamethasone, which effectively reduces HMB. [**(A)**, right] During endometrial repair, pharmacological stabilisation of HIF1A may prevent the delayed repair thought to occur in those with AUB-E. Correcting TGFB deficiency with nanoparticles carrying soluble TGFB may be another strategy to improve the symptom of AUB-E. **(B)** In adenomyosis, AUB-A may be improved via endocrine manipulation. The selective progesterone modulator UPA and GnRH antagonists have shown promising results in clinical trials by targeting PR and/or ER in adenomyotic deposits and the eutopic endometrium. Therapeutic strategies focused on compensating the overactivation of NFκB (using NFκB inhibitors) and TF (using anti-platelet therapy) observed both in the eutopic endometrium and adenomyotic lesions may also be beneficial in AUB-A. AUB, abnormal uterine bleeding; AUB-E, AUB of endometrial origin; AUB-A, AUB due to adenomyosis; FL, functional layer; BL, basal layer; Myo, myometrium; TNF-α, tumour necrosis factor; siRNA, small interfering RNA; TGF-β, transforming growth factor beta; HIF-1α, hypoxia inducible factor 1 alpha; NFκB, nuclear factor kappa B; TF, Tissue factor; UPA, ulipristal acetate; GnRH-ant, gonadotropin releasing hormone antagonists; ER, oestrogen receptor; PR, progesterone receptor.

Current treatment options for AUB remain suboptimal, as highlighted by the results of the Royal College of Obstetricians and Gynaecologists (RCOG) UK HMB audit (89). In this audit of patients attending hospital gynaecology clinics (*n* = 8183), 37% of women remained “unhappy” or “very unhappy” with their on-going HMB symptoms (89). There is a clear need for new and improved medical treatment strategies for AUB (Figure 1).

### Emerging Treatments: Endocrine Manipulation

Ulipristal acetate (UPA) is a selective progesterone receptor modulator with prevailing inhibitory effects on the progesterone receptor that may be used as a treatment for AUB-L (90). The safety and effectiveness of UPA has been assessed in three clinical studies under the PEARL (PGL4001 Efficacy Assessment in Reduction of Symptoms Due to Uterine Leiomyomata) programme, where UPA administration resulted in effective control of AUB (90). Interestingly, UPA reduces AUB as effectively as the GnRH agonist Leuprorelin but without hypoestrogenic side effects (90). Some adverse effects associated with UPA treatment have been noted, such as weight gain and fatigue and recently potential concerns regarding negative liver effects have been highlighted (91).

The effectiveness of UPA in AUB-L management raises the possibility of trialling this therapeutic strategy in those with AUB-E and AUB-A (Figure 1B). In an observational study in women with adenomyosis, treatment with UPA for 12 weeks reduced symptoms of AUB (92). A phase II, randomised, double-blind controlled trial with UPA 10 mg/day for 3 months in patients with adenomyosis has been registered (NCT02587000). UPA has been shown to impact the endometrium at a cellular and molecular level. In particular, it has been shown to reduce to expression of endometrial steroid metabolising enzymes. A reduction in *HSD11B1*, known to metabolise cortisol, was observed (93). As previously discussed, cortisol is thought to play a role in regulating the menstrual inflammatory response. These findings support the potential use of UPA in patients with AUB-E.

Aromatase (CYP19A1*)* has been detected in the endometrium of women with endometriosis, adenomyosis and leiomyomas but not in normal endometrium and has therefore been proposed as a potential therapeutic target (94). A small prospective randomised controlled study compared the oral aromatase inhibitor letrozole and the subcutaneous GnRH agonist Goserelin in the treatment of adenomyosis. In both groups, a similar reduction in uterine volume and adenomyoma volume was observed and two patients in the letrozole group became pregnant during treatment (95).

GnRH-antagonists have also emerged as a new strategy for improving AUB (Figure 1B). As opposed to conventional GnRH agonists, GnRH antagonists directly inhibit LH and FSH synthesis. This mechanism of action skips the initial surge of the pituitary hormones aforementioned that occurs with GnRH agonist treatment. Moreover, the dose of GnRH-antagonist used may be titrated to allow only partial suppression of oestrogen. This reduces the potential consequences of a hypoestrogenic state and additional add-back HRT may not be required. As an example, the GnRH-antagonist Elagolix significantly reduced HMB in patients with uterine fibroids and coexisting adenomyosis (96).

### Emerging Treatments: Breakdown and Inflammation

As discussed above, inflammation and NFκB signalling pathways may play a crucial role in the initiation of endometrial breakdown, with overactivation resulting in AUB (Figure 1B). Andrographolide is an active ingredient from the plant *Andrographis paniculata* and has been used for many years in traditional Chinese medicine for the treatment of inflammatory disorders. It has been shown to suppress NFκB activation (97). In a pre-clinical model of adenomyosis, the intragastric administration of andrographolide reduced myometrial leukocyte infiltration as well as adenomyosis-derived pain (98). The impact of andrographolide treatment on menstrual blood loss was not reported in this study and merits further examination.

A more refined strategy may be to target downstream mediators of NFκB that have been shown to be dysregulated in AUB, such as TNF (48). At present, there is a wide range of anti-TNF antibodies which effectively block the biological function of TNF (99). However, systemic administration of these drugs is not without side effects, given the key role of TNF against pathogen invasion (99). Therefore, local endometrial anti-TNF delivery may be beneficial. As an example, polymer-coated nanoparticles containing small interfering (si)RNA targeting TNF have been proven to be successful in a mouse model of rheumatoid arthritis, reducing TNF levels in serum and arthritic joints (100). This technology has the potential to be adapted for endometrial disorders once the endometrial source of TNF is confirmed (Figure 1A).

Cortisol deficiency may also play a key role in the development of AUB-E via dysregulation of the endometrial repair processes. The role of low dose dexamethasone as a treatment to reduce HMB has been demonstrated in a recent response-adaptive randomised placebo-controlled dose-finding parallel group trial (DexFEM) (101). This trial showed that dexamethasone 1.8 mg once daily over 5 days in the mid-secretory phase reduced menstrual blood loss when compared with placebo. However, 75% of the participants in the dexamethasone group reported adverse events, compared with 58% of those taking placebo. Nonetheless, this study demonstrated that dexamethasone may provide an effective treatment option for AUB in women who wish to avoid ovarian hormone based treatments (Figure 1A).

### Emerging Treatments: Limiting Blood Loss

Treatments that correct aberrations identified in the endometrial coagulation system of those with AUB have potential as novel therapeutics or preventative strategies for AUB-A (Figure 1B). In a mouse model of adenomyosis, treatment with Ozagrel, a platelet aggregation inhibitor, supressed myometrial infiltration and improved adenomyosis related pain (102). As highlighted by the authors of this proof-of-concept study, concerns remain about the possible risk of haemorrhage associated with such an anti-platelet therapy. While this study adds to our understanding of the role of activated platelets in the pathogenesis of adenomyosis, further examination of the safety profile of such medications is required before translation to clinical trials.

### Emerging Treatments: Endometrial Repair and Regeneration

Markers of hypoxia have been detected in the human endometrium at menstruation (38) and physiological hypoxia has been shown to be important in endometrial repair during simulated menses in the mouse (37). Pharmacological stabilisation of HIF1 in a mouse model of delayed endometrial repair rescued the phenotype and improved repair, indicating that targeting the hypoxia pathway may be a valid approach in the treatment of AUB (37) (Figure 1A). HIF1A stabilisation is not an unfamiliar therapeutic strategy in the treatment of non-gynaecological disorders. Pharmacological stabilisation of HIF1A has proven to be effective and safe in the treatment of anaemia in chronic kidney disease (103, 104). In addition, a small HIF1A stabiliser has showed promise in accelerating diabetic wound healing in different pre-clinical models (105). These compounds may have therapeutic benefits in the endometrium, which is amenable to local and intermittent treatment.

Correcting the partial deficiency of TGFB during menstruation that has been detected in the endometrium of those with AUB-E may present another valid therapeutic strategy for AUB (Figure 1A). Due to the numerous pleiotropic effects of TGFB as a cytokine, the systemic administration of a soluble version is far from ideal (106). However, recent literature offers different strategies for local delivery of soluble mediators. Using inert biodegradable nanoparticles loaded with TGFB, McHugh et al. achieved T cell-specific delivery both *in vitro* and in animal models (107). This technique has the potential to be applied in the endometrial environment, perhaps via transvaginal administration, targeting endometrial, immune and/or vascular endothelial cells. Liposomes are another alternative nanoparticle with good pre-clinical results (108) that could be of use in TGFB delivery if designed for local endometrial action.

## A Future Model for Management of AUB

Despite advances in understanding of the mechanisms and pathological processes that lead to AUB, many treatments remain broad spectrum and generic. Often the focus is on AUB symptom control rather than specific diagnosis and targeted treatment.

Achieving an accurate diagnosis starts with focused history taking and clinical examination and should involve reference to FIGO AUB System 1 (nomenclature) and System 2 (classification; PALM-COEIN) (1). This directs further relevant investigations and personalises management. For example, identifying and understanding a patient's wish for fertility, previous experience with treatments and assessment of the size, position, regularity, mobility, and tenderness of the uterus will help aid clinicians to tailor management options specifically suited to that patient and their diagnosis. Further research to determine how patient demographics such as age, BMI and physical activity influence menstrual blood loss and response to treatment may also help to select more effective treatments for individuals (109).

To assist clinical diagnosis, bedside tests that identify the presence of structural uterine disorders and/or identify the specific cause(s) of endometrial dysfunction would be highly valuable. For example, the ability to identify if a women with AUB-E had aberrations in endometrial hypoxia, inflammation and/or coagulation at the time of endometrial sampling would facilitate personalised medicine and correction of the specific underlying defect causing AUB. These tests may also assist in the selection of appropriate investigations such as ultrasound or hysteroscopy.

The gold standard for diagnosis of adenomyosis is histopathological confirmation of eutopic endometrium within the myometrium. However, the use of transvaginal ultrasound (TVUS) in achieving accurate diagnosis of adenomyosis is highlighted in a systematic review (46). Of the 8 studies included, TVUS 2D and TVUS 3D were shown to be effective methods for diagnosis of adenomyosis with pooled sensitivity of 84 and 89%, and pooled specificity of 64 and 56%, respectively (46). These findings support the use of imaging in obtaining an accurate diagnosis but focused research to improve the specificity of such methods would have significant clinical and scientific benefits.

Several biomarkers for adenomyosis have also been proposed. For example, proteomic analysis has shown that moesin, a cytoskeletal adaptor protein, is higher in the endometrium of those with adenomyosis vs. controls (110). This finding may also help our understanding of the development of adenomyosis as moesin expression is correlated with the extent of invasiveness seen in some tumours, such as gastric adenocarcinoma (110). Full discussion of all emerging biomarkers for AUB is not possible in this review but such findings highlight the potential for improving the non-invasive diagnosis of this debilitating symptom.

Improved diagnosis of structural and non-structural causes of AUB will not only direct treatment but will facilitate research into the pathogenesis of specific diagnoses (e.g., AUB-E/A) and reveal new therapeutic targets and preventative strategies to improve the lives of those who suffer from AUB.

## Conclusions

Furthering our understanding of menstrual physiology confirms that there is a complex interplay of endocrine, immune, haemostatic and vascular regulatory functions. This complexity is mirrored in the pathological aberrations that may occur in each of these pathways, resulting in AUB. We have demonstrated that current medical management of AUB is suboptimal by highlighting poor satisfaction rates and the lack of specific, targeted treatments. Emerging treatments offer the promise of more specific targeting of the underlying pathology causing AUB-E and AUB-A. Accurate diagnosis of the underlying cause of AUB should be a clinical and research priority. Future research should also focus on developing therapies which have direct actions against the pathological processes which have been demonstrated to result in AUB-E and AUB-A. Focused clinical assessment and imaging techniques may help toward this goal but the development of non-invasive biomarkers would be a significant step toward improving management. These efforts would facilitate the development of personalised, effective, acceptable treatments to improve the lives of those who experience AUB.

## Author Contributions

MW and RM-A wrote the manuscript. JM, MW, and RM-A planned and edited the manuscript. All authors have read and approved the final version of this manuscript.

## Funding

Some aspects of studies described herein were undertaken in the MRC Centre for Reproductive Health which was funded by MRC Centre Grants G1002033 and MR/N022556/1. This work was also in part funded by Wellcome Trust Grant 209589/Z/17/Z and Tenovus Scotland.

## Conflict of Interest

The authors declare that the research was conducted in the absence of any commercial or financial relationships that could be construed as a potential conflict of interest.

## Publisher's Note

All claims expressed in this article are solely those of the authors and do not necessarily represent those of their affiliated organizations, or those of the publisher, the editors and the reviewers. Any product that may be evaluated in this article, or claim that may be made by its manufacturer, is not guaranteed or endorsed by the publisher.

## References

[1] Munro MG Critchley HO Broder MS Fraser IS. FIGO classification system (PALM-COEIN) for causes of abnormal uterine bleeding in nongravid women of reproductive age. Int J Gynaecol Obstet. (2011) 113:3–13. 10.1016/j.ijgo.2010.11.011 21345435

[2] Munro MG Critchley HOD Fraser IS. The two FIGO systems for normal and abnormal uterine bleeding symptoms and classification of causes of abnormal uterine bleeding in the reproductive years: 2018 revisions. Int J Gynaecol Obstet. (2018) 143:393–408. 10.1002/ijgo.12666 30198563

[3] Vilos GA Lefebvre G Graves GR. Guidelines for the management of abnormal uterine bleeding: these guidelines have been reviewed by the clinical practice gynaecology and the reproductive endocrinology infertility committees, and approved by executive and council of the society of obstetricians and gynaecologists of Canada. J SOGC. (2001) 23:704–9. 10.1016/S0849-5831(16)31463-X

[4] Liu Z Doan QV Blumenthal P Dubois RW. A systematic review evaluating health-related quality of life, work impairment, and health-care costs and utilization in abnormal uterine bleeding. Value Health. (2007) 10:183–94. 10.1111/j.1524-4733.2007.00168.x 17532811

[5] Royal College of Obstreticians and Gynaecologists (RCOG), London School of Hygiene and Tropical Medicine (LSHTM) MORI I. National Heavy Menstrual Bleeding Audit: Second Annual Report (2012).

[6] Whitaker L Critchley HOD. Abnormal uterine bleeding. Best Pract Res Clin Obstet Gynaecol. (2016) 34:54–65. 10.1016/j.bpobgyn.2015.11.012 26803558 PMC4970656

[7] Pennant ME Mehta R Moody P Hackett G Prentice A Sharp SJ. Premenopausal abnormal uterine bleeding and risk of endometrial cancer. BJOG. (2017) 124:404–11. 10.1111/1471-0528.14385 27766759 PMC5297977

[8] Davies J Kadir RA. Endometrial haemostasis and menstruation. Rev Endocr Metab Disord. (2012) 13:289–99. 10.1007/s11154-012-9226-4 23180227

[9] Ferenczy A Bertrand G Gelfand MM. Proliferation kinetics of human endometrium during the normal menstrual cycle. Am J Obstet Gynecol. (1979) 133:859–67. 10.1016/0002-9378(79)90302-8 434029

[10] Finn CA. Implantation, menstruation and inflammation. Biol Rev. (1986) 61:313–28. 10.1111/j.1469-185X.1986.tb00657.x 3542071

[11] King AE Critchley HOD Kelly RW. The NF-κB pathway in human endometrium and first trimester decidua. Mol Hum Reprod. (2001) 7:175–83. 10.1093/molehr/7.2.175 11160844

[12] Kelly RW King AE Critchley HO. Cytokine control in human endometrium. Reproduction. (2001) 121:3–19. 10.1530/rep.0.1210003 11226025

[13] Evans J Salamonsen LA. Decidualized human endometrial stromal cells are sensors of hormone withdrawal in the menstrual inflammatory cascade. Biol Reprod. (2014) 90:14. 10.1095/biolreprod.113.108175 24227758

[14] Armstrong GM Maybin JA Murray AA Nicol M Walker C Saunders PTK. Endometrial apoptosis and neutrophil infiltration during menstruation exhibits spatial and temporal dynamics that are recapitulated in a mouse model. Sci Rep. (2017) 7:17416. 10.1038/s41598-017-17565-x 29234102 PMC5727295

[15] Evans J Salamonsen LA. Inflammation, leukocytes and menstruation. Rev Endocr Metab Disord. (2012) 13:277–88. 10.1007/s11154-012-9223-7 22865231

[16] Azlan A Salamonsen LA Hutchison J Evans J. Endometrial inflammasome activation accompanies menstruation and may have implications for systemic inflammatory events of the menstrual cycle. Hum Reprod. (2020) 35:1363–76. 10.1093/humrep/deaa065 32488243

[17] Guo H Callaway JB Ting JPY. Inflammasomes: mechanism of action, role in disease, and therapeutics. Nat Med. (2015) 21:677–87. 10.1038/nm.3893 26121197 PMC4519035

[18] Salamonsen L Woolley D. Menstruation: induction by matrix metalloproteinases and inflammatory cells. J Reproduct Immunol. (1999) 44:1–27. 10.1016/S0165-0378(99)00002-9 10530758

[19] Wu B Chen X He B Liu S Li Y Wang Q. ROS are critical for endometrial breakdown via NF-κB–COX-2 signaling in a female mouse menstrual-Like Model. Endocrinology. (2014) 155:3638–48. 10.1210/en.2014-1029 24926822

[20] Rae MT Niven D Ross A Forster T Lathe R Critchley HO. Steroid signalling in human ovarian surface epithelial cells: the response to interleukin-1alpha determined by microarray analysis. J Endocrinol. (2004) 183:19–28. 10.1677/joe.1.05754 15525570

[21] McDonald SE Henderson TA Gomez-Sanchez CE Critchley HO Mason JI. 11Beta-hydroxysteroid dehydrogenases in human endometrium. Mol Cell Endocrinol. (2006) 248:72–8. 10.1016/j.mce.2005.12.010 16406280

[22] Maybin JA Boswell L Young VJ Duncan WC Critchley HOD. Reduced transforming growth factor-β activity in the endometrium of women with heavy menstrual bleeding. J Clin Endocrinol Metab. (2017) 102:1299–308. 10.1210/jc.2016-3437 28324043 PMC5460733

[23] Sanjabi S Zenewicz LA Kamanaka M Flavell RA. Anti-inflammatory and pro-inflammatory roles of TGF-beta, IL-10, and IL-22 in immunity and autoimmunity. Curr Opin Pharmacol. (2009) 9:447–53. 10.1016/j.coph.2009.04.008 19481975 PMC2755239

[24] Thiruchelvam U Maybin JA Armstrong GM Greaves E Saunders PTK Critchley HOD. Cortisol regulates the paracrine action of macrophages by inducing vasoactive gene expression in endometrial cells. J Leukoc Biol. (2016) 99:1165–71. 10.1189/jlb.5A0215-061RR 26701134 PMC4952012

[25] Maybin JA Thiruchelvam U Madhra M Saunders PTK Critchley HOD. Steroids regulate CXCL4 in the human endometrium during menstruation to enable efficient endometrial repair. J Clin Endocrinol Metab. (2017) 102:1851–60. 10.1210/jc.2016-3604 28323919 PMC5470763

[26] Serhan CN Chiang N Van Dyke TE. Resolving inflammation: dual anti-inflammatory and pro-resolution lipid mediators. Nat Rev Immunol. (2008) 8:349–61. 10.1038/nri2294 18437155 PMC2744593

[27] Macdonald LJ Boddy SC Denison FC Sales KJ Jabbour HNA. role for lipoxin A4 as an anti-inflammatory mediator in the human endometrium. Reproduction. (2011) 142:345–52. 10.1530/REP-11-0021 21555360 PMC3139491

[28] Jones RL Kelly RW Critchley HO. Chemokine and cyclooxygenase-2 expression in human endometrium coincides with leukocyte accumulation. Hum Reprod. (1997) 12:1300–6. 10.1093/humrep/12.6.1300 9222021

[29] Critchley HOD Kelly RW Brenner RM Baird DT. The endocrinology of menstruation - a role for the immune system. Clin Endocrinol. (2001) 55:701–10. 10.1046/j.1365-2265.2001.01432.x 11895208

[30] Sugino N Karube-Harada A Taketani T Sakata A Nakamura Y. Withdrawal of ovarian steroids stimulates prostaglandin F2alpha production through nuclear factor-kappaB activation via oxygen radicals in human endometrial stromal cells: potential relevance to menstruation. J Reprod Dev. (2004) 50:215–25. 10.1262/jrd.50.215 15118249

[31] Salamonsen LA Marsh MM Findlay JK. Endometrial endothelin: regulator of uterine bleeding and endometrial repair. Clin Exp Pharmacol Physiol. (1999) 26:154–7. 10.1046/j.1440-1681.1999.03012.x 10065338

[32] Palta S Saroa R Palta A. Overview of the coagulation system. Indian J Anaesth. (2014) 58:515–23. 10.4103/0019-5049.144643 25535411 PMC4260295

[33] Gelety TJ Chaudhuri G. Haemostatic mechanism in the endometrium: role of cyclo-oxygenase products and coagulation factors. Br J Pharmacol. (1995) 114:975–80. 10.1111/j.1476-5381.1995.tb13300.x 7780653 PMC1510305

[34] Markee JE. Menstruation in intraocular endometrial transplants in the rhesus monkey. Contrib Embryol. (1940) (177):219–308. 98044 10.1016/0002-9378(78)90119-9

[35] Fan X Krieg S Kuo CJ Wiegand SJ Rabinovitch M Druzin ML. VEGF blockade inhibits angiogenesis and reepithelialization of endometrium. FASEB J. (2008) 22:3571–80. 10.1096/fj.08-111401 18606863 PMC2537439

[36] Cousins FL Murray AA Scanlon JP Saunders PTK. Hypoxyprobe™ reveals dynamic spatial and temporal changes in hypoxia in a mouse model of endometrial breakdown and repair. BMC Res Notes. (2016) 9:30. 10.1186/s13104-016-1842-8 26780953 PMC4717617

[37] Maybin JA Murray AA Saunders PTK Hirani N Carmeliet P Critchley HOD. Hypoxia and hypoxia inducible factor-1α are required for normal endometrial repair during menstruation. Nat Commun. (2018) 9:295. 10.1038/s41467-017-02375-6 29362355 PMC5780386

[38] Reavey JJ Walker C Nicol M Murray AA Critchley HOD Kershaw LE. Markers of human endometrial hypoxia can be detected in vivo and ex vivo during physiological menstruation. Human Reproduction. (2021) 36:941–50. 10.1093/humrep/deaa379 33496337 PMC7970728

[39] Critchley HO Osei J Henderson TA Boswell L Sales KJ Jabbour HN. Hypoxia-inducible factor-1alpha expression in human endometrium and its regulation by prostaglandin E-series prostanoid receptor 2 (EP2). Endocrinology. (2006) 147:744–53. 10.1210/en.2005-1153 16282352

[40] Coudyzer P Lemoine P Jordan BF Gallez B Galant C Nisolle M. Hypoxia is not required for human endometrial breakdown or repair in a xenograft model of menstruation. FASEB J. (2013) 27:3711–9. 10.1096/fj.13-232074 23729593

[41] Semenza Gregg L. Hypoxia-inducible factors in physiology and medicine. Cell. (2012) 148:399–408. 10.1016/j.cell.2012.01.021 22304911 PMC3437543

[42] Maybin JA Barcroft J Thiruchelvam U Hirani N Jabbour HN Critchley HOD. The presence and regulation of connective tissue growth factor in the human endometrium. Hum Reprod. (2012) 27:1112–21. 10.1093/humrep/der476 22328559 PMC3303491

[43] Maybin JA Battersby S Hirani N Nikitenko LL Critchley HOD Jabbour HN. The expression and regulation of Adrenomedullin in the human endometrium: a candidate for endometrial repair. Endocrinology. (2011) 152:2845–56. 10.1210/en.2010-1256 21558311 PMC3192419

[44] Maybin JA Hirani N Jabbour HN Critchley HOD. Novel roles for hypoxia and prostaglandin E2 in the regulation of IL-8 during endometrial repair. Am J Pathol. (2011) 178:1245–56. 10.1016/j.ajpath.2010.11.070 21356375 PMC3047791

[45] Peric H Fraser IS. The symptomatology of adenomyosis. Best Pract Res Clin Obstet Gynaecol. (2006) 20:547–55. 10.1016/j.bpobgyn.2006.01.006 16515888

[46] Andres MP Borrelli GM Ribeiro J Baracat EC Abrão MS Kho RM. Transvaginal ultrasound for the diagnosis of Adenomyosis: systematic review and meta-analysis. J Minim Invasive Gynecol. (2018) 25:257–64. 10.1016/j.jmig.2017.08.653 28864044

[47] Livingstone M Fraser IS. Mechanisms of abnormal uterine bleeding. Hum Reprod Update. (2002) 8:60–7. 10.1093/humupd/8.1.60 11866241

[48] Malik S Day K Perrault I Charnock-Jones DS Smith SK. Reduced levels of VEGF-A and MMP-2 and MMP-9 activity and increased TNF-α in menstrual endometrium and effluent in women with menorrhagia. Hum Reprod. (2006) 21:2158–66. 10.1093/humrep/del089 16585124

[49] Hayden MS Ghosh S. Regulation of NF-κB by TNF family cytokines. Semin Immunol. (2014) 26:253–66. 10.1016/j.smim.2014.05.004 24958609 PMC4156877

[50] Smith OP Jabbour HN Critchley HO. Cyclooxygenase enzyme expression and E series prostaglandin receptor signalling are enhanced in heavy menstruation. Hum Reprod. (2007) 22:1450–6. 10.1093/humrep/del503 17264103 PMC2694303

[51] Park H Kim S-H Cho YM Ihm HJ Oh YS Hong SH. Increased expression of nuclear factor kappa-B p65 subunit in adenomyosis. Obstet Gynecol Sci. (2016) 59:123–9. 10.5468/ogs.2016.59.2.123 27004203 PMC4796082

[52] Li B Chen M Liu X Guo S-W. Constitutive and tumor necrosis factor-α-induced activation of nuclear factor-κB in adenomyosis and its inhibition by andrographolide. Fertil Steril. (2013) 100:568–77. 10.1016/j.fertnstert.2013.04.028 23706331

[53] Sotnikova N Antsiferova I Malyshkina A. Cytokine network of eutopic and ectopic endometrium in women with adenomyosis. Am J Reprod Immunol. (2002) 47:251–5. 10.1034/j.1600-0897.2002.01040.x 12069392

[54] Nie J Lu Y Liu X Guo SW. Immunoreactivity of progesterone receptor isoform B, nuclear factor kappaB, and IkappaBalpha in adenomyosis. Fertil Steril. (2009) 92:886–9. 10.1016/j.fertnstert.2009.01.084 19296948

[55] Li C Chen R Jiang C Chen L Cheng Z. Correlation of LOX-5 and COX-2 expression with inflammatory pathology and clinical features of adenomyosis. Mol Med Rep. (2019) 19:727–33. 10.3892/mmr.2018.9618 30387822

[56] Rae M Mohamad A Price D Hadoke PWF Walker BR Mason JI. Cortisol Inactivation by 11β-Hydroxysteroid dehydrogenase-2 may enhance endometrial angiogenesis via reduced thrombospondin-1 in heavy menstruation. J Clin Endocrinol Metab. (2009) 94:1443–50. 10.1210/jc.2008-1879 19158196

[57] Marsh MM Malakooti N Taylor NH Findlay JK Salamonsen LA. Endothelin and neutral endopeptidase in the endometrium of women with menorrhagia. Hum Reprod. (1997) 12:2036–40. 10.1093/humrep/12.9.2036 9363725

[58] Smith SK Abel MH Kelly RW Baird DT. Prostaglandin synthesis in the endometrium of women with ovular disfunctional uterine bleeding. BJOG Int J Obstet Gynaecol. (1981) 88:434–42. 10.1111/j.1471-0528.1981.tb01009.x 7225303

[59] Chen Y-J Li H-Y Chang Y-L Yuan C-C Tai L-K Lu KH. Suppression of migratory/invasive ability and induction of apoptosis in adenomyosis-derived mesenchymal stem cells by cyclooxygenase-2 inhibitors. Fertil Steril. (2010) 94:1972–9. 10.1016/j.fertnstert.2010.01.070 20227073

[60] Philibert P Déjardin S Pirot N Pruvost A Nguyen AL Bernex F. In the mouse, prostaglandin D2 signalling protects the endometrium against adenomyosis. Mol Hum Reprod. (2021) 27:gaab029. 10.1093/molehr/gaab029 33851217

[61] Gleeson N Devitt M Sheppard BL Bonnar J. Endometrial fibrinolytic enzymes in women with normal menstruation and dysfunctional uterine bleeding. Br J Obstet Gynaecol. (1993) 100:768–71. 10.1111/j.1471-0528.1993.tb14272.x 8399019

[62] Yang B Gu N Shi S Zhang C Chen L Ouyang J. Immunoreactivity of Plasminogen activator inhibitor 1 and its correlation with dysmenorrhea and lesional fibrosis in adenomyosis. Reprod sci. (2021) 28:2378–86. 10.1007/s43032-021-00513-6 33683668 PMC8289782

[63] Liu X Nie J Guo S-W. Elevated immunoreactivity to tissue factor and its association with dysmenorrhea severity and the amount of menses in adenomyosis. Hum Reprod. (2010) 26:337–45. 10.1093/humrep/deq311 21115504

[64] Hao M Liu X Guo S-W. Adenomyosis in mice resulting from mechanically or thermally induced endometrial–myometrial interface disruption and its possible prevention. Reprod Biomed Online. (2020) 41:925–42. 10.1016/j.rbmo.2020.07.023 32921577

[65] Lu Q Sun D Shivhare SB Hou H Bulmer JN Innes BA. Transforming growth factor (TGF) β and endometrial vascular maturation. Front Cell Dev Biol. (2021) 9:640065. 10.3389/fcell.2021.640065 33898426 PMC8063037

[66] Mints M Hultenby K Zetterberg E Blomgren B Falconer C Rogers R. Wall discontinuities and increased expression of vascular endothelial growth factor-A and vascular endothelial growth factor receptors 1 and 2 in endometrial blood vessels of women with menorrhagia. Fertil Steril. (2007) 88:691–7. 10.1016/j.fertnstert.2006.11.190 17336974

[67] Abberton KM Taylor NH Healy DL Rogers PAW. Vascular smooth muscle cell proliferation in arterioles of the human endometrium. Hum Reprod. (1999) 14:1072–9. 10.1093/humrep/14.4.1072 10221243

[68] Abberton KM Healy DL Rogers PAW. Smooth muscle alpha actin and myosin heavy chain expression in the vascular smooth muscle cells surrounding human endometrial arterioles. Hum Reprod. (1999) 14:3095–100. 10.1093/humrep/14.12.3095 10601102

[69] Biswas Shivhare S Bulmer JN Innes BA Hapangama DK Lash GE. Endometrial vascular development in heavy menstrual bleeding: altered spatio-temporal expression of endothelial cell markers and extracellular matrix components. Hum Reprod. (2018) 33:399–410. 10.1093/humrep/dex378 29309596

[70] Liu X Shen M Qi Q Zhang H Guo S-W. Corroborating evidence for platelet-induced epithelial-mesenchymal transition and fibroblast-to-myofibroblast transdifferentiation in the development of adenomyosis†. Hum Reprod. (2016) 31:734–49. 10.1093/humrep/dew018 26908845

[71] Wang J Deng X Yang Y Yang X Kong B Chao L. Expression of GRIM-19 in adenomyosis and its possible role in pathogenesis. Fertil Steril. (2016) 105:1093–101. 10.1016/j.fertnstert.2015.12.019 26769301

[72] Li T Li YG Pu DM. Matrix metalloproteinase-2 and−9 expression correlated with angiogenesis in human adenomyosis. Gynecol Obstet Invest. (2006) 62:229–35. 10.1159/000094426 16837781

[73] Huang TS Chen YJ Chou TY Chen CY Li HY Huang BS. Oestrogen-induced angiogenesis promotes adenomyosis by activating the Slug-VEGF axis in endometrial epithelial cells. J Cell Mol Med. (2014) 18:1358–71. 10.1111/jcmm.12300 24758741 PMC4124020

[74] Heavy Menstrual Bleeding: Assessment And Management. National Institute for Health and Care Excellence (NICE) (2018). 29634173

[75] Lethaby A Wise MR Weterings MAJ Bofill Rodriguez M Brown J. Combined hormonal contraceptives for heavy menstrual bleeding. Cochrane Database Syst Rev. (2019) 2: CD000154. 10.1002/14651858.CD000154.pub3 30742315 PMC6369862

[76] UK Medical Eligibility Criteria for Contraceptive Use (UKMEC). The Faculty of Sexual & Reproductive Healthcare of the Royal College of Obstetricians & Gynaecologists (FSRH) (2016).

[77] Roberts TE Tsourapas A Middleton LJ Champaneria R Daniels JP Cooper KG. Hysterectomy, endometrial ablation, and levonorgestrel releasing intrauterine system (Mirena) for treatment of heavy menstrual bleeding: cost effectiveness analysis. BMJ. (2011) 342:d2202. 10.1136/bmj.d2202 21521730 PMC3082380

[78] Bhattacharya S Middleton LJ Tsourapas A Lee AJ Champaneria R Daniels JP. Hysterectomy, endometrial ablation and Mirena® for heavy menstrual bleeding: a systematic review of clinical effectiveness and cost-effectiveness analysis. Health Technol Assess. (2011) 15:1–252. 10.3310/hta15190 21535970 PMC4781434

[79] Shaaban OM Ali MK Sabra AM Abd El Aal DE. Levonorgestrel-releasing intrauterine system versus a low-dose combined oral contraceptive for treatment of adenomyotic uteri: a randomized clinical trial. Contraception. (2015) 92:301–7. 10.1016/j.contraception.2015.05.015 26071673

[80] Faculty of Sexual & Reproductive Healthcare (FSRH). Intrauterine Contraception. (2015) (accessed September 2019).

[81] Lethaby A Vollenhoven B Sowter M. Pre-operative GnRH analogue therapy before hysterectomy or myomectomy for uterine fibroids. Cochrane Database Syst Rev. (2001) 2:Cd000547. 10.1002/14651858.CD000547 11405968

[82] Takeuchi H Kobori H Kikuchi I Sato Y Mitsuhashi N. A prospective randomized study comparing endocrinological and clinical effects of two types of GnRH agonists in cases of uterine leiomyomas or endometriosis. J Obstet Gynaecol Res. (2000) 26:325–31. 10.1111/j.1447-0756.2000.tb01334.x 11147718

[83] Cetin NN Karabacak O Korucuoglu U Karabacak N. Gonadotropin-releasing hormone analog combined with a low-dose oral contraceptive to treat heavy menstrual bleeding. Int J Gynaecol Obstet. (2009) 104:236–9. 10.1016/j.ijgo.2008.10.032 19062012

[84] Lethaby A Duckitt K Farquhar C. Non-steroidal anti-inflammatory drugs for heavy menstrual bleeding. Cochrane Database Syst Rev. (2013) 1:Cd000400. 10.1002/14651858.CD000400.pub3 23440779

[85] Fraser IS Pearse C Shearman RP Elliott PM McIlveen J Markham R. Efficacy of mefenamic acid in patients with a complaint of menorrhagia. Obstet Gynecol. (1981) 58:543–51. 10.1097/00006254-198205000-00015 7029369

[86] Hall P Maclachlan N Thorn N Nudd MW Taylor CG Garrioch DB. Control of menorrhagia by the cyclo-oxygenase inhibitors naproxen sodium and mefenamic acid. Br J Obstet Gynaecol. (1987) 94:554–8. 10.1111/j.1471-0528.1987.tb03150.x 3304401

[87] Mäkäräinen L Ylikorkala O. Primary and myoma-associated menorrhagia: role of prostaglandins and effects of ibuprofen. Br J Obstet Gynaecol. (1986) 93:974–8. 10.1111/j.1471-0528.1986.tb08019.x 3533137

[88] Matteson KA Rahn DD Wheeler TL Casiano E Siddiqui NY Harvie HS. Nonsurgical management of heavy menstrual bleeding: a systematic review. Obstet Gynecol. (2013) 121:632–43. 10.1097/AOG.0b013e3182839e0e 23635628 PMC4414119

[89] Royal College of Obstreticians and Gynaecologists (RCOG) London School of Hygiene and Tropical Medicine (LSHTM) MORI I. National Heavy Menstrual Bleeding Audit: Third Annual Report (2013).

[90] Powell M Dutta D. Esmya(®) and the PEARL studies: a review. Womens Health. (2016) 12:544–8. 10.1177/1745505717692591 29334010 PMC5373264

[91] Hong YH Han SJ Lee D Kim SK Jee BC. Adverse symptoms during short-term use of ulipristal acetate in women with uterine myomas and/or adenomyosis. J Obstet Gynaecol Res. (2019) 45:865–70. 10.1111/jog.13917 30675965

[92] Gracia M Alcalà M Ferreri J Rius M Ros C Saco MA. Ulipristal Acetate improves clinical symptoms in women with Adenomyosis and uterine myomas. J Minim Invasive Gynecol. (2018) 25:1274–80. 10.1016/j.jmig.2018.04.002 29626678

[93] Chodankar RR Murray A Nicol M Whitaker LHR Williams ARW Critchley HOD. The endometrial response to modulation of ligand-progesterone receptor pathways is reversible. Fertil Steril. (2021) 116:882–95. 10.1016/j.fertnstert.2021.02.008 33865567

[94] Kitawaki J Noguchi T Amatsu T Maeda K Tsukamoto K Yamamoto T. Expression of aromatase cytochrome P450 protein and messenger ribonucleic acid in human endometriotic and adenomyotic tissues but not in normal endometrium. Biol Reprod. (1997) 57:514–9. 10.1095/biolreprod57.3.514 9282984

[95] Badawy AM Elnashar AM Mosbah AA. Aromatase inhibitors or gonadotropin-releasing hormone agonists for the management of uterine adenomyosis: a randomized controlled trial. Acta Obstet Gynecol Scand. (2012) 91:489–95. 10.1111/j.1600-0412.2012.01350.x 22229256

[96] Muneyyirci-Delale O Archer DF Owens CD Barnhart KT Bradley LD Feinberg E. Efficacy and safety of elagolix with add-back therapy in women with uterine fibroids and coexisting adenomyosis. F S Rep. (2021) 2:338–46. 10.1016/j.xfre.2021.05.004 34553161 PMC8441572

[97] Xia YF Ye BQ Li YD Wang JG He XJ Lin X. Andrographolide attenuates inflammation by inhibition of NF-kappa B activation through covalent modification of reduced cysteine 62 of p50. J Immunol. (2004) 173:4207–17. 10.4049/jimmunol.173.6.4207 15356172

[98] Mao X Wang Y Carter AV Zhen X Guo SW. The retardation of myometrial infiltration, reduction of uterine contractility, and alleviation of generalized hyperalgesia in mice with induced adenomyosis by levo-tetrahydropalmatine (l-THP) and andrographolide. Reprod Sci. (2011) 18:1025–37. 10.1177/1933719111404610 21493954

[99] Li P Zheng Y Chen X. Drugs for autoimmune inflammatory diseases: from small molecule compounds to anti-TNF biologics. Front Pharmacol. (2017) 8:460. 10.3389/fphar.2017.00460 28785220 PMC5506195

[100] Lee SJ Lee A Hwang SR Park J-S Jang J Huh MS. TNF-α gene silencing using polymerized siRNA/thiolated glycol chitosan nanoparticles for rheumatoid arthritis. Mol Ther. (2014) 22:397–408. 10.1038/mt.2013.245 24145554 PMC3916041

[101] Warner P Whitaker LHR Parker RA Weir CJ Douglas A Hansen CH. Low dose dexamethasone as treatment for women with heavy menstrual bleeding: a response-adaptive randomised placebo-controlled dose-finding parallel group trial (DexFEM). EBioMedicine. (2021) 69:103434. 10.1016/j.ebiom.2021.103434 34218053 PMC8261537

[102] Zhu B Chen Y Shen X Liu X Guo S-W. Anti-platelet therapy holds promises in treating adenomyosis: experimental evidence. Reprod Biol Endocrinol. (2016) 14:66. 10.1186/s12958-016-0198-1 27724926 PMC5057470

[103] Akizawa T Macdougall IC Berns JS Bernhardt T Staedtler G Taguchi M. Long-term efficacy and safety of molidustat for anemia in chronic kidney disease: DIALOGUE Extension studies. Am J Nephrol. (2019) 49:271–80. 10.1159/000499111 30852574 PMC6518868

[104] Zheng Q Yang H Sun L Wei R Fu X Wang Y. Efficacy and safety of HIF prolyl-hydroxylase inhibitor vs epoetin and darbepoetin for anemia in chronic kidney disease patients not undergoing dialysis: a network meta-analysis. Pharmacol Res. (2020) 159:105020. 10.1016/j.phrs.2020.105020 32561478

[105] Li G Ko CN Li D Yang C Wang W Yang GJ. A small molecule HIF-1α stabilizer that accelerates diabetic wound healing. Nat Commun. (2021) 12:3363. 10.1038/s41467-021-23448-7 34099651 PMC8184911

[106] Calabresi PA Fields NS Maloni HW Hanham A Carlino J Moore J. Phase 1 trial of transforming growth factor beta 2 in chronic progressive MS. Neurology. (1998) 51:289–92. 10.1212/WNL.51.1.289 9674825

[107] McHugh MD Park J Uhrich R Gao W Horwitz DA Fahmy TM. Paracrine co-delivery of TGF-β and IL-2 using CD4-targeted nanoparticles for induction and maintenance of regulatory T cells. Biomaterials. (2015) 59:172–81. 10.1016/j.biomaterials.2015.04.003 25974747 PMC5997248

[108] Abe T Yamada H Nakajima H Kikuchi T Takaishi H Tadakuma T. Repair of full-thickness cartilage defects using liposomal transforming growth factor-beta1. J Orthop Sci. (2003) 8:92–101. 10.1007/s007760300016 12560894

[109] Reavey JJ Walker C Murray AA Brito-Mutunayagam S Sweeney S Nicol M. Obesity is associated with heavy menstruation that may be due to delayed endometrial repair. J Endocrinol. (2021) 249:71–82. 10.1530/JOE-20-0446 33836495 PMC8052524

[110] Ohara R Michikami H Nakamura Y Sakata A Sakashita S Satomi K. Moesin overexpression is a unique biomarker of adenomyosis. Pathol Int. (2014) 64:115–22. 10.1111/pin.12148 24698421

